# Longitudinal hippocampal subfield development associated with psychotic experiences in young people

**DOI:** 10.1038/s41398-024-02746-w

**Published:** 2024-01-20

**Authors:** Aisling O’Neill, Niamh Dooley, Darren Roddy, Colm Healy, Eleanor Carey, Thomas Frodl, Erik O’Hanlon, Mary Cannon

**Affiliations:** 1 Department of Psychology, St Patrick’s Mental Health Services, Dublin, Ireland; 2 Department of Psychiatry, RCSI University of Medicine and Health Sciences, St Stephens Green, Dublin, Ireland; 3 Trinity College Institute of Neuroscience, Trinity College Dublin, Dublin, Ireland; 4 Department of Medicine, University College Dublin, Dublin, Ireland; 5 Klinik für Psychiatrie, Psychotherapie und Psychosomatik, Uniklinik RWTH Aachen, Aachen, Germany

**Keywords:** Psychiatric disorders, Neuroscience, Physiology

## Abstract

Hippocampal volumetric reductions are observed across the psychosis spectrum, with interest in the localisation of these reductions within the hippocampal subfields increasing. Deficits of the CA1 subfield in particular have been implicated in the neuropathophysiology of psychotic disorders. Investigating the trajectory of these abnormalities in healthy adolescents reporting sub-threshold psychotic experiences (PE) can provide insight into the neural mechanisms underlying psychotic symptoms without the potentially confounding effects of a formal disorder, or antipsychotic medication. In this novel investigation, a sample of 211 young people aged 11-13 participated initially in the Adolescent Brain Development study. PE classification was determined by expert consensus at each timepoint. Participants underwent neuroimaging at 3 timepoints, over 6 years. 78 participants with at least one scan were included in the final sample; 33 who met criteria for a definite PE at least once across all the timepoints (PE group), and 45 controls. Data from bilateral subfields of interest (CA1, CA2/3, CA4/DG, presubiculum and subiculum) were extracted for Linear Mixed Effects analyses. Before correction, subfield volumes were found to increase in the control group and decrease in the PE group for the right CA2 and CA2/3 subfields, with moderate to large effect sizes (*d* = −0.61, and *d* = −0.79, respectively). Before correction, right subiculum and left presubiculum volumes were reduced in the PE group compared to controls, regardless of time, with moderate effect sizes (*d* = −0.52, and *d* = −0.59, respectively). However, none of these effects survived correction. Severity of symptoms were not associated with any of the noted subfields. These findings provide novel insight to the discussion of the role of hippocampal subfield abnormalities in the pathophysiology underlying psychotic experiences.

## Introduction

The prevalence of subthreshold psychotic experiences amongst young people up to the age of 24 in the general population is thought to be as high as 8.1% [1]. However, despite not having a formal diagnosis, these individuals are still at increased risk for developing psychosis, additional psychopathology, and can display subtle neurocognitive impairments [2–6]. Moderate neuroanatomical changes have also been observed in individuals with PE [7–13], although these are much more subtle than in individuals with psychotic disorders [14–22].

The hippocampus plays a crucial role in a range of cognitive functions including memory, learning, executive functioning and emotion processing, while also playing a primary role in regulation of the stress response—all of which are impaired to varying degrees in individuals with psychosis [23, 24]. As such, hippocampal abnormalities in particular have been strongly implicated in psychosis literature [20, 25, 26]. We recently identified longitudinal whole volume deficits in the hippocampi of adolescents who report PE, using the adolescent brain development (ABD) cohort [12, 27, 28]. Associations have also been observed between deficits in hippocampal size and growth and expression of schizotypy traits in adolescents in the community [29, 30], reflecting the findings of our previous work. These findings are also largely in keeping with studies of individuals in the prodromal stages of psychosis [31–33], potentially implicating hippocampal reductions as a marker of psychosis vulnerability.

Early studies of hippocampal anatomy have focused on the whole structure or larger divisions (e.g. anterior vs posterior structures) [34]. However, as neuroimaging technologies have improved, interest has grown in the smaller, cytoarchitecturally distinct—yet highly interconnected—subfields of the hippocampus, specifically the cornu ammonis (CA) 1–4, dentate gyrus (DG) and subiculum complex. Indeed, authors of meta-analyses exploring whole volume hippocampal changes in the prodromal stages of psychosis suggest that the lack of subfield data may result in subtle deficits being overlooked [33, 35].

In their recent review, Hu et al. suggest that volume deficits in the CA1 are the most prominent abnormalities observed in those in the prodromal stages of psychosis [34]. It has been argued that these deficits are more severe in those who transition to psychosis [36, 37], and that the alterations may spread throughout the hippocampus and beyond as the illness progresses [17, 19, 38–40]. However, the localisations and trajectories of these deficits within the functionally discrete subfields of the hippocampus in individuals who report PE have yet to be explored. Furthermore, current research is largely limited to cross-sectional studies of treatment seeking individuals (who have either received a diagnosis of a psychotic disorder or are experiencing prodromal symptoms of psychosis). As such, longitudinal investigations involving adolescents and young people who report PE will provide novel and important insights into the developmental trajectories of these regions in the absence of illness or medications.

Thus, the aim of this study is to investigate the developmental trajectory of the hippocampal subfields spanning approximately 6 years of adolescence, in young people who report PE. Bilateral hippocampal subfields of interest (ROIs) were based on the protocols of previous studies [17, 24, 34]; and included CA1, CA2/CA3, CA4/DG, subiculum and presubiculum. Based on the findings of these studies, we predict that there will be significant deficits in the volumetric development of the CA1 subfield in young people who report PE. This study will provide novel insight into the mechanisms underlying psychotic psychopathology and psychosis vulnerability, without the potentially confounding effects of a formal diagnosis. This is the first study to examine hippocampal subfields longitudinally in young people with psychotic experiences.

## Methods and materials

Ethics committee approval was obtained from the Beaumont Hospital Medical Ethics committee and the Trinity College Department of Psychology Ethics committee.

### Participants

A sample of 211 young people between the ages of 11 and 13 years old were recruited from primary schools in Dublin and Kildare, Ireland, as part of the Adolescent Brain Development (ABD) study [28]. The initial 20% of young people recruited to the ABD study was enriched at a rate of 2:1 for adolescents with a score of 2 or more on the Adolescent Psychotic Symptom Screener [28, 41]. All 211 were invited to participate in the initial neuroimaging arm of the ABD study (baseline) that took place 1–3 years (mean 2 years) after the original interview. 100 participants with no contraindications to structural magnetic resonance imaging (sMRI) completed this baseline scan. Of these 100, 69 participants returned for follow-up 1 (2 years later). For the final timepoint of the neuroimaging arm (follow-up 2, 4 years later), 55 of the original participants returned and were scanned. Overall, 78 participants who had been scanned at least once over the three imaging timepoints were included in the sample here; 33 who met criteria for a definite PE at least once across all the timepoints (PE group), and 45 who did not meet criteria for a PE at any timepoint (control group).

None of the participants in either group met the criteria for a formal psychotic disorder or were taking antipsychotic medication, and none had any history of neurological disorder (e.g. epilepsy). Written parental consent and participant assent were obtained before the study began, and at each follow-up for participants under the age of 18. Participant consent was obtained after the age of 18.

### Clinical measures

All participants attended a diagnostic clinical interview with trained raters (additional recruitment and interview details outlined in Kelleher et al. [28]). Adolescents and parents/guardians were interviewed separately, both answering the same questions about the adolescent, using the Schedule for Affective disorders and schizophrenia for school-age children (K-SADS) [42] at baseline and follow-up 1. At follow-up 2, participants were interviewed, using the Structured Clinical Interview for DSM-5 (SCID) [43] (additional details outlined in Carey et al. [44]). The psychosis section of K-SADS and the SCID were supplemented by additional questions from the SOCRATES instrument, which was devised to systematically assess the presence of PE in youth populations [45]. Additional symptom data was collected at baseline and follow-up 2 using the Structured Interview for psychosis-risk syndromes (SIPS) [46]. Parents/guardians were not interviewed for follow-ups 1 and 2. All interviews were reviewed by a consensus committee (two psychiatrists and a psychologist) in order to confirm PE classification at each timepoint (further details in Supplementary Material).

Data were also collected for factors strongly associated with PE and psychosis, and which have also been implicated in hippocampal abnormalities in the general population and in psychiatric populations other than psychosis [47, 48]. As part of the K-SADS at the baseline assessment, childhood adversity data was collected and treated as a dichotomous variable (i.e. yes/no childhood adversity reported) (details in Supplementary Material). During the final assessment, lifetime DSM 5 diagnoses (excluding simple phobias) were recorded, and also treated as a dichotomous variable (i.e. yes/no DSM 5 diagnosis reported). Data relating to lifetime DSM 5 diagnoses were only available for participants that returned for the interview at follow-up 2 (PE = 20, controls = 22) (breakdown of diagnoses in the Supplementary Material).

### Structural MRI data acquisition

Whole-brain sMRI data were acquired for each participant using the same 3 T magnetic resonance imaging system (Philips Achieva, Philips Medical Systems Netherland BV), in Trinity College Institute of Neuroscience, Dublin. High resolution T1 weighted images were acquired with a fast field echo 3-D transverse sequence, using the following parameters: TE/TR = 8.4/3.9 ms; flip angle = 8°; 256 × 256 matrix; 180 × 0.9 mm slices; FoV = 230. Scan duration was 5:44 min.

### Pre-processing of structural data

Hippocampal reconstruction, and volumetric segmentation and parcellation was performed with the FreeSurfer image analysis suite version 6.0 and developmental toolbox for longitudinal analysis features (http://surfer.nmr.mgh.harvard.edu/). The technical details of these procedures are described in previous publications [49]. The FreeSurfer hippocampal subfield longitudinal processing stream was used to extract reliable longitudinal volumes and thickness estimates [50]. Systematic inspection of the data included visual inspection of the images, quality control of the imaging data following the ENIGMA Consortium Imaging Protocols (https://enigma.ini.usc.edu/protocols/imaging-protocols/), and outlier identification. Outliers were defined using the 1st and 3rd Quartiles (Q1 and Q3) and the interquartile range (IQR), where volumes that fell below Q1 − 1.5 IQR or above Q3 + 1.5 IQR were considered extreme outliers. Outlier detection identified three participants, one with observable structural abnormalities (a PE participant), and two whose data was corrupted during the acquisition step (one PE participant, and one control participant). All three participants identified through outlier detection were removed.

### Statistical analysis

All statistical analyses were performed using R 4.0.2 [51], and the lme4 v1.1.23 package [52]. Bilateral hippocampal subfields of interest (ROIs) were based on the protocols of previous studies [17, 24, 34]. These subfields were as follows: subiculum, presubiculum, CA1, CA2/CA3, CA4/DG.

In subfield analyses, the CA4 is included in the DG region, as it is considered functionally part of the DG structure [53]. Similarly, the small CA2 region is combined with CA3 in subfield analyses, due to the difficulty in distinguishing the two [49, 54].

Our statistical approach follows our previous procedure [12]. Briefly, linear mixed effects modelling (LME) was used for analysis of all the longitudinal volumetric data, with separate models computed for each ROI. LME methods were chosen as they allow the inclusion of participants with missing data points, and varying intervals between measurements, and because they combine the components of fixed effects, random effects and repeated-measures within a single model [55, 56].

In the current study, fixed effects of interest were group and interactions between Group × Time. Fixed effects covariates were intracranial volume (ICV), time (in months since baseline), age (in months) at baseline, gender and handedness. DSM 5 lifetime diagnosis was also included as a fixed effect, as a significant difference was observed between the groups for this variable (see Results: Demographics; and Table 1). As no significant differences were observed for adversity at baseline (see Results: Demographics; and Table 1), this was not included in the models.

**Table 1:** Participant socio-demographic information.

	PE (*n* = 33)	Controls (*n* = 45)	Statistics	95% CI
	Mean (SD)			
Number of scans	2.27 (0.8) 1 scan: 7 2 scans: 10 3 scans: 16	2.15 (0.8) 1 scan: 11 2 scans: 16 3 scans: 18	*p* = 0.52	(−0.48; 0.25)
Age in months at BL	167.56 (14.45)	170.64 (16.6)	*p* = 0.4	(−4.17; 10.33)
Age in months at FU1	193.25 (14.89)	195.72 (17.35)	*p* = 0.58	(−6.55; 11.5)
Age in months at FU2	237.1 (18.45)	244.86 (21.58)	*p* = 0.22	(−4.82; 20.35)
Gender (%male)	66.7	42.2	*p* = 0.033^a^	(0.02; 0.47)
Handedness (%right)	97	88.9	*p* = 0.26	(−0.046; 0.17)
Socioeconomic status	2.2 (0.96)	2.17 (0.91)	*X*^*2*^ = 0.038, p = 1	
Adversity at BL (%yes)	66.7	46.7	*p* = 0.079	(−0.42; 0.024)
DSM5 diagnosis ever* (%yes)	(*n* = 25) 76	(*n* = 23) 30.4	*p* = 0.001^a^	(−0.72; −0.19)
SIPS scores*	*(n* = *25)* BL: 27.24 (13.45) FU2: 30.62 (16.06)	*(n* = *23)* BL: 4.86 (4.21) FU2: 16.32 (6.35)	BL: *p* < 0.001 FU2: *p* < 0.001	BL: (−28.45; −16.29) FU2: (−28.71; −16.03)
PE recurrence across timepoints	1 timepoint: 20 2 timepoints: 6 3 timepoints: 7	N/A		

Socioeconomic status was established via highest parental occupation level, categorised as follows: 1 = professional work, 2 = managerial and technical work, 3 = nonmanual work, 4 = skilled manual work, 5 = semiskilled work, 6 = unskilled work, 7 = unemployed. *PE* psychotic experiences group, *SD* standard deviation, *SIPS* structured interview for psychosis-risk syndromes.

^a^Significant *p* value.

*Sample limited to those who attended follow-up 2.

DSM5 diagnosis ever data were only available for those participants who returned at FU2 (PE = 25, controls = 20).

Subjects were treated as random effects, to account for within person correlations (in brain volumes) inherent in longitudinal analyses, and individual variations including random intercept (e.g. normal volumetric differences in the ROI at baseline) and random slope (e.g. normal variations in individual rates of change). Age at baseline, age at scan and ICV were mean centred for the analysis.

Top-down model selection followed an established protocol [56]. This involved fitting a forced entry model with the greatest number of fixed effects and random effects (full model), which are then removed in a backwards fashion. Likelihood ratio tests and Akaike Information Criterion Corrected (AICc) were used to compare models and determine best fit for each ROI. False discovery rate (FDR) procedures were used to correct for multiple ROI comparisons [57].

### Symptoms

For any subfields that displayed significant uncorrected differences between the PE and control groups, any overall associations between subfield volume and total SIPS scores in the PE group were also explored via linear mixed effects models (using the same approach outlined above).

### Additional analyses

Additional demographic analyses exploring group differences relating to sampling, selection, attrition biases and multicollinearity are reported in the Supplementary Material.

## Results

### Demographics

Participant demographic information is summarised in Table 1. Mean age at baseline (BL) was 167.56 months (14 years; SD = 14.45 months) in the PE group and 170.64 months (14.2 years; SD = 16.6 months) in the control group. Mean age at Follow-up 1 (FU1) was 193.25 months (SD = 14.89 months)/16.1 years (PE) and 195.72 months (SD = 17.35 months)/16.31 years (controls). Mean age at second follow-up (FU2) was 237.1 months (SD = 18.45 months)/19.76 years (PE) and 244.86 months (SD = 21.58 months)/20.4 years (controls). All participants were antipsychotic naïve across all timepoints. At baseline, none of the 78 included participants were taking psychiatric medications. By the final follow-up, three participants reported taking anti-depressant medications. All three of these participants were in the PE group. Of the 33 participants who met the criteria for PE at some point, 20 participants met the criteria for a PE at one timepoint, 6 participants met the criteria at two timepoints, and 7 participants met the criteria at three timepoints. In the PE group, 7 participants had one scan, 10 had two scans and 16 had three scans. In the control group, 11 participants had one scan, 16 had two scans and 18 had three scans. There were no significant differences between the groups in terms of number of scans, age at BL, FU1 or FU2, handedness, socioeconomic status, or adversity at baseline. Significantly more of the PE participants were male (*p* = 0.033). Significantly more of the PE participants had received a DSM5 diagnosis at some point in their lives than the control group (*p* = 0.001).

### Model selection process

#### Random effects structure

The model including slope was not found to be appropriate for any of the volumes. Thus, for all the bilateral hippocampal subfields, only the intercept was included in the random effects structure.

#### Fixed effects structure

The full model (including all higher order fixed effects of interest) was found to be most appropriate (significantly better than the null model), and subsequently fitted for the right CA1 and CA2/3 subfields. The reduced model (no interaction effect, i.e. excluding Group × Time) was found to be most appropriate for the left presubiculum and right subiculum volumes. For all other volumes, the null model was most appropriate, indicating no significant effects on volume due to the fixed measures of interest.

### Between group differences

The significant mixed model analyses results for the covariates of interest are shown in Table 2 (full table for all covariates are displayed in the Supplementary material, Table S4).

**Table 2:** Results of the mixed effects model analyses for hippocampal subfield volumes in the PE and control participants.

	Group		Group × time since BL	
GM volumes of interest	*B* (SE)	*t*, *p*	*B* (SE)	*t*, *p*
L presubiculum	−23.69 (10)	*t* = −2.37, *p* = 0.021^a^ (FDR = 0.1)	N/A	N/A
R CA1	11.08 (21.02)	*t* = 0.53, *p* = 0.6	−0.37 (0.18)	*t* = −2.082, *p* = 0.043^a^ (FDR = 0.21)
R CA2/3	11.04 (8.64)	*t* = 1.16, *p* = 0.25	−0.22 (0.085)	*t* = −2.58, *p* = 0.013^a^ (FDR = 0.065^b^)
R subiculum	−24.07 (11.43)	*t* = −2.1, *p* = 0.039^a^ (FDR = 0.19)	N/A	N/A

*PE* psychotic experience, *L* left, *R* right, *B* estimate of the fixed effect coefficient, *SE* standard error, *BL* baseline, *FDR* false discovery rate, *N/A* not applicable—for left presubiculum and right subiculum, the fitted model did not include the Group × Time interaction effect. FDR-corrected *p* is reported for variables of interest significant at the uncorrected level; all other values displayed are uncorrected.

^a^Significant *p* value.

^b^Trend level effect.

**Fig. 1:**
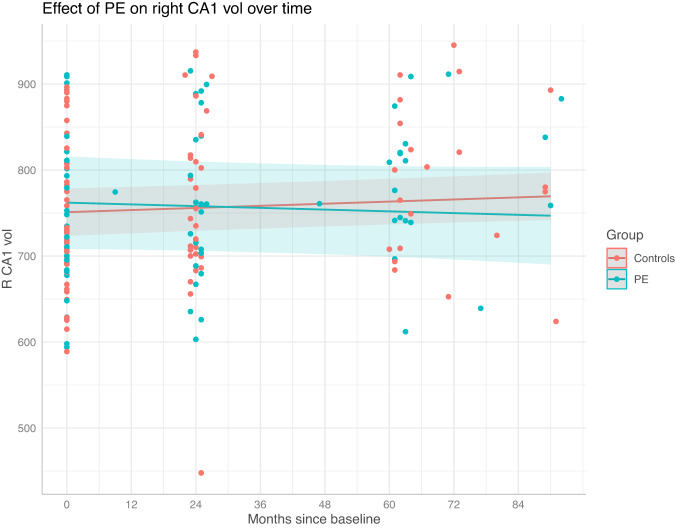
Right CA1 development in the participants with and without PE from baseline to follow-up 2. The shaded region shows the 95% confidence interval. A Group × Time interaction effect was observed, such that right CA1 volume increased in the control group, and remained relatively stable in the PE group. This effect did not survive false discovery rate correction.

**Fig. 2:**
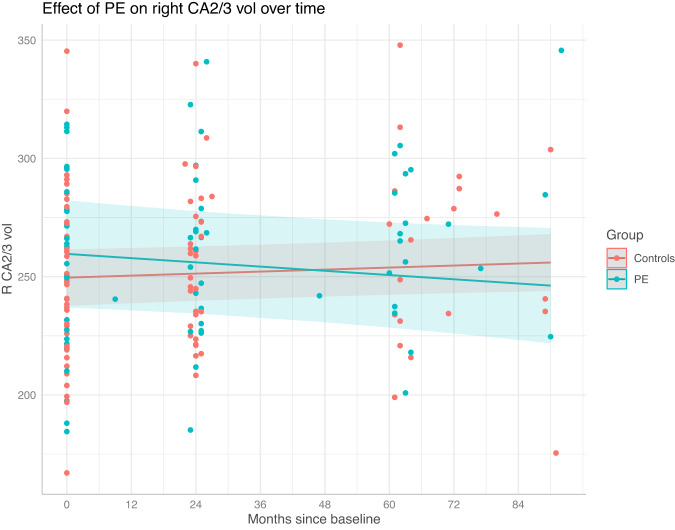
Right CA2/3 development in the participants with and without PE from baseline to follow-up 2. The shaded region shows the 95% confidence interval. A Group × Time interaction effect was observed, such that right CA2/3 volume increased in the control group, and remained relatively stable in the PE group. This effect did not survive false discovery rate correction.

Before correction, the right CA1 and CA2/3 subfields displayed significant Group × Time interactions (CA1: *p* = 0.043, FDR *p* = 0.21, cohen’s *d* = −0.61; CA2/3: *p* = 0.013, FDR *p* = 0.065, cohen’s *d* = −0.79). This effect was such that for both subfields, the volume increased in the control group, and decreased in the PE group (Figs. 1 and 2). Neither of these effects survived correction, although the CA2/3 interaction effect had a large effect size, and was approaching significance.

**Fig. 3:**
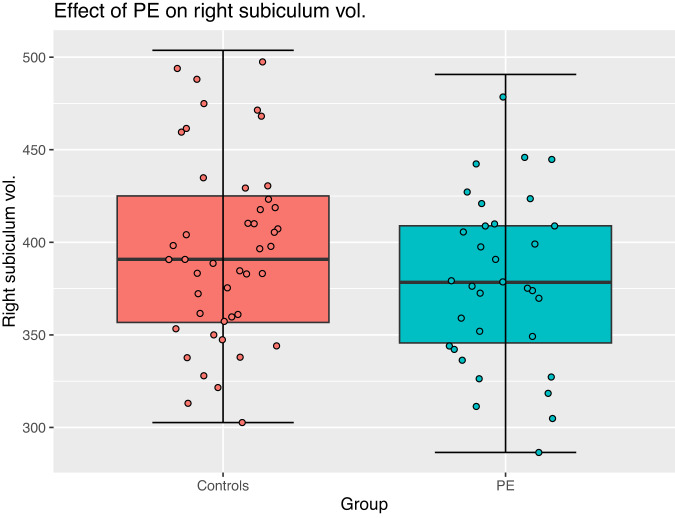
Box-plot of the right subiculum volume in participants with and without PE. The horizontal line inside the boxes represents the median. Lower and upper box boundaries represent the 25th and 75th percentiles, respectively. The vertical extending lines denote the most extreme values within the 1.5 interquartile range of the 25th and 75th percentile of each group. The error bars represent the 95% confidence interval. Scatterplot points represent the average volume across time for each participant. A Group effect was observed, with the control group displaying greater right subiculum volumes compared to the PE group, regardless of time. This effect did not survive false discovery rate correction.

**Fig. 4:**
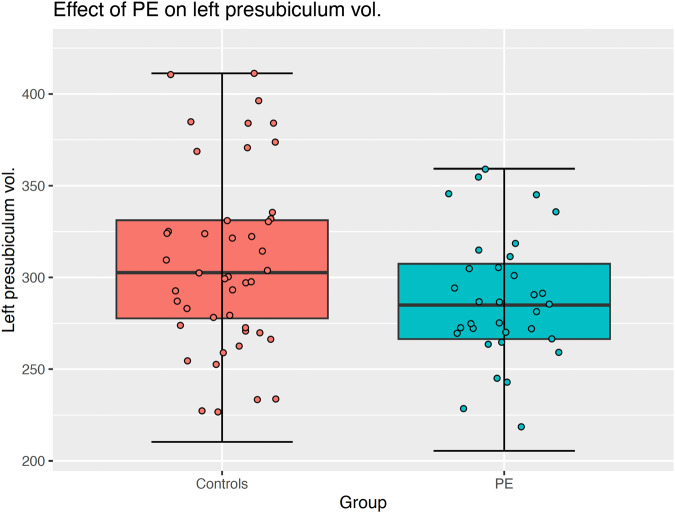
Box-plot left presubiculum volume in participants with and without PE. The horizontal line inside the boxes represents the median. Lower and upper box boundaries represent the 25th and 75th percentiles, respectively. The vertical extending lines denote the most extreme values within the 1.5 interquartile range of the 25th and 75th percentile of each group. The error bars represent the 95% confidence interval. Scatterplot points represent the average volume across time for each participant. A Group effect was observed, with the control group displaying greater left presubiculum volumes compared to the PE group, regardless of time. This effect did not survive false discovery rate correction.

In terms of group effects (regardless of time), the PE group displayed smaller right subiculum volumes (*p* = 0.039, FDR = 0.19, cohen’s *d* = −0.52) (Fig. 3); and smaller left presubiculum volumes (*p* = 0.021, FDR *p* = 0.1, cohen’s *d* = −0.59) (Fig. 4). Neither of these effects survived correction.

No significant differences were observed for any of the other subfields (i.e. right presubiculum, left subiculum, bilateral CA4/DG).

### Symptoms

None of the subfields identified in the between groups analyses were significantly associated with SIPS scores over time. However, these results should be interpreted with caution, as SIPS data were only available for the baseline and follow-up 2 timepoints.

## Discussion

In this study, we explored the development of the hippocampal subfields, over 6 years, in young people who report PE. Before FDR correction, we identified differences in the right CA1 and CA2/3 subfields, where the volumes of both regions decreased over time in the PE group, compared to an increase over time in the control group. We also identified reduced volumes in the PE group regardless of time in the left presubiculum, and in the right subiculum. Although these differences did not survive correction, the group differences in the left presubiculum and right subiculum, and interaction effects for the right CA1 and right CA2/3 subfields all displayed moderate to large effect sizes, with the right CA2/3 interaction effect approaching significance after correction. Given the non-clinical nature of the sample, any PE related differences between the groups are likely to be very subtle. The moderate to large effect sizes suggest that the differences observed here may still have practical significance [58], or may reach statistical significance in a larger sample [59].

Given the findings of previous research that describe reductions in the CA1 subfield initially, with more widely spread subfield volumetric reductions as severity of symptoms increases across the psychosis spectrum (i.e. sub-threshold to chronic) [17, 19, 24, 34, 38, 39, 60], we predicted that there would be significant PE related deficits in the CA1 subfield. However, the differences observed in this cohort were not limited to CA1, with the strongest effect observed in the right CA2/3 subfield. Indeed, our findings for the right CA2/3 and subiculum complex were generally more in line with those of people with a diagnosis of psychotic disorder, as reviewed by Haukvik et al. [24] and Hu et al. [34]. Nonetheless, these results are not entirely unprecedented, as reduced CA2/3 and CA1 volumes have also been observed in individuals at ultra-high-risk for psychosis [61]. Furthermore, lower presubiculum volumes have been associated with higher resting cortisol levels in clinical high risk for psychosis and first episode psychosis [62, 63], suggesting an early stress related role for the subiculum complex in psychosis. Overall, these findings support those of our previous investigation of adolescent global grey matter structures which only identified PE related differences in the right hippocampus [12]. Building on these findings, the lack of significant FDR corrected differences within the subfields investigated here may alternatively indicate that PE related differences are diffused across the hippocampus, rather than specific to any one area. Following this perspective, within subfield differences may be too subtle to survive correction in isolation, but cumulatively they are observable.

The hippocampus is known to play a critical role in memory, with memory impairments during the premorbid phase argued to be predictive of future transition to psychosis [64]. However, the specific role of the hippocampus subfields in psychosis is not yet clearly understood. One potential hypothesis suggests that glutamate dysregulation in the CA1 subfield leads to hyperactivity in the hippocampus complex, eliciting attenuated psychotic symptoms [39]. Following this argument, in individuals for whom symptoms persist into chronic illness, the initial CA1 dysfunction is succeeded by the spread of dysfunction across further subfields in the hippocampus [19, 38, 39, 60]. Another possibly complementary theory considers the specific role of the CA3 subfield in memory, suggesting that abnormal CA3 neuronal activity may underlie exaggerated pattern completion during memory processing. This in turn may contribute to poor discrimination and incorrect association of memories, ultimately laying a foundation for psychotic symptoms [65, 66]. In individuals with psychosis, the subiculum complex volume has been associated positively with metacognition (i.e. thinking about thinking) [67], and negatively with over-confidence [68]. Combined with a potential role in episodic memory, subiculum complex deficits may contribute to poor insight and inaccurate error monitoring across the psychosis spectrum [60, 67, 68].

A potential explanation for the subfield growth trajectories observed in the PE group is that they are underpinned by a developmental delay. This is supported by neurocognitive findings in psychosis spectrum young people, which show significant developmental lags in domains including complex cognition and social cognition [69], and by our previous finding of PE related delays in the widespread development of functional connectivity during adolescence [70]. Such developmental delays may result from, for example, disordered synaptic pruning and grey matter maturation during adolescence, in line with the neural efficiency threshold model [71]. In keeping with this theory, in the current study, a decrease in hippocampal volumes was observed in the PE group over time, in contrast with the control group and typical hippocampal development observed during adolescence [72].

It is notable that there was a lack of association between the symptoms data and the subfield volumes in the current study. Although the symptom data was limited here to total scores, similar findings have also been noted in previous studies in first episode psychosis [73]. This may reflect the subtlety of the volumetric differences in these samples.

What differentiates those for whom symptoms persist, and those whose experiences are transient remains unclear. A recent meta-analysis of whole hippocampal volumetric studies identified no significant effect of whole hippocampal volume on transition risk, although right hippocampal volumes appeared to predict transition at the trend level [33]. Studies of hippocampal subfields largely agree that CA1 is implicated to some extent in conversion to psychosis [24, 34]. This agreement diverges regarding the timing of these CA1 abnormalities; i.e. whether they are present at baseline [17, 37], or only observable after conversion [19, 36]. Indeed, in non-clinical populations who report psychotic experiences, underlying mechanisms indicating healthy functioning may be more apparent in other dimensions, e.g. adaptive integration of experiences [74]. A recent qualitative study involving a separate subset of the ABD cohort found that individuals’ appraisals of their psychotic experiences greatly influenced how pathological these experiences were [75]. Due to the moderate numbers of participants who experienced PE recurrence in this cohort, it was not possible to conduct analyses comparing transient vs persistent PE, or rates of transition to psychosis in adulthood. Future studies of PE should explore neurophysiological differences in relation to additional subjective features which may indicate the healthy expression of psychotic experiences.

A potential limitation of the study is the restricted data relating to symptom severity over time, lifetime DSM-5 diagnoses, and childhood adversity (symptom severity and DSM-5 diagnoses data were only available for participants who took part in follow-up 2, and childhood adversity data were only collected at baseline). While no significant effects were observed for the subfields and these covariates, it is possible that this approach could overlook associations between the covariates, brain morphology and PE. Additionally, the reconceptualization of psychotic disorders as occurring on a spectrum, similar to those spectrum phenotypes covering autism and addiction, is becoming more accepted [76]. In the current study, the categorisation of weak/strong PEs from the original protocol (details in the Supplementary Material) limits the implementation of a spectral analysis of this sort. The “weak” category represents experiences not convincing enough to be classified as a definite psychotic experience. Participants classified as having only “weak” PE were excluded from the analysis, as their inclusion could lead to misleading results owing to mislabelling of non-PEs. In the future, more nuanced approaches to categorisation of experiences should be considered, to better represent the spectrum of experiences.

The findings are strengthened by the use of LME statistics, which allows the inclusion of all valid participant datapoints (i.e. all valid participants were included, regardless of whether they attended all timepoints). Additionally, none of the participants were receiving antipsychotic treatment. This is a key strength, as few studies have explored the potentially confounding effects of antipsychotic medications [24, 34]. Further methodological strengths include the population-based youth sample, the consensus-based criteria for PEs, the well-matched control participants, the neuroimaging data all being acquired from the same scanner, and the use of the FreeSurfer longitudinal processing stream. However, despite it being frequently used, the accuracy of the version of Freesurfer used has been questioned [24, 77], and as such, future studies should take this into consideration.

Overall, the findings from the current study emphasise the importance of the hippocampus across the psychosis spectrum. These findings add novel insights into the hippocampal subfield volumetry underlying psychosis symptomology, and further may implicate the CA1 and CA2/3 subfields as part of the core substrates underlying psychosis pathophysiology.

### Supplementary information

Supplementary Material

## References

[1] Sullivan SA, Kounali D, Cannon M, David AS, Fletcher PC, Holmans P (2020). A population-based cohort study examining the incidence and impact of psychotic experiences from childhood to adulthood, and prediction of psychotic disorder. Am J Psychiatry.

[2] Healy C, Brannigan R, Dooley N, Coughlan H, Clarke M, Kelleher I (2019). Childhood and adolescent psychotic experiences and risk of mental disorder: a systematic review and meta-analysis. Psychol Med.

[3] Kaymaz N, Drukker M, Lieb R, Wittchen HU, Werbeloff N, Weiser M (2012). Do subthreshold psychotic experiences predict clinical outcomes in unselected non-help-seeking population-based samples? A systematic review and meta-analysis, enriched with new results. Psychol Med.

[4] Kelleher I, Clarke MC, Rawdon C, Murphy J, Cannon M (2013). Neurocognition in the extended psychosis phenotype: performance of a community sample of adolescents with psychotic symptoms on the MATRICS neurocognitive battery. Schizophr Bull.

[5] Kelleher I, Devlin N, Wigman JT, Kehoe A, Murtagh A, Fitzpatrick C (2014). Psychotic experiences in a mental health clinic sample: implications for suicidality, multimorbidity and functioning. Psychol Med.

[6] Nishida A, Sasaki T, Nishimura Y, Tanii H, Hara N, Inoue K (2010). Psychotic-like experiences are associated with suicidal feelings and deliberate self-harm behaviors in adolescents aged 12-15 years. Acta Psychiatr Scand.

[7] Satterthwaite TD, Wolf DH, Calkins ME, Vandekar SN, Erus G, Ruparel K (2016). Structural brain abnormalities in youth with psychosis spectrum symptoms. JAMA Psychiatry.

[8] Okada N, Yahata N, Koshiyama D, Morita K, Sawada K, Kanata S (2018). Abnormal asymmetries in subcortical brain volume in early adolescents with subclinical psychotic experiences. Transl Psychiatry.

[9] Jacobson S, Kelleher I, Harley M, Murtagh A, Clarke M, Blanchard M (2010). Structural and functional brain correlates of subclinical psychotic symptoms in 11-13 year old schoolchildren. Neuroimage.

[10] Orr JM, Turner JA, Mittal VA (2014). Widespread brain dysconnectivity associated with psychotic-like experiences in the general population. Neuroimage Clin.

[11] Dooley N, O’Hanlon E, Healy C, Adair A, McCandless C, Coppinger D (2020). Psychotic experiences in childhood are associated with increased structural integrity of the left arcuate fasciculus—a population-based case-control study. Schizophr Res.

[12] O’Neill A, Dooley N, Healy C, Carey E, Roddy D, Frodl T (2022). Longitudinal gray matter development associated with psychotic experiences in young people. Biol Psychiatry Glob Open Sci.

[13] O’Hanlon E, Leemans A, Kelleher I, Clarke MC, Roddy S, Coughlan H (2015). White matter differences among adolescents reporting psychotic experiences: A population-based diffusion magnetic resonance imaging study. JAMA Psychiatry.

[14] Allen P, Azis M, Modinos G, Bossong MG, Bonoldi I, Samson C (2017). Increased resting hippocampal and basal ganglia perfusion in people at ultra high risk for psychosis: replication in a second cohort. Schizophr Bull.

[15] Heckers S, Konradi C (2015). GABAergic mechanisms of hippocampal hyperactivity in schizophrenia. Schizophr Res.

[16] Mathew I, Gardin TM, Tandon N, Eack S, Francis AN, Seidman LJ (2014). Medial temporal lobe structures and hippocampal subfields in psychotic disorders: findings from the Bipolar-Schizophrenia Network on Intermediate Phenotypes (B-SNIP) study. JAMA Psychiatry.

[17] McHugo M, Armstrong K, Roeske MJ, Woodward ND, Blackford JU, Heckers S (2020). Hippocampal volume in early psychosis: a 2-year longitudinal study. Transl Psychiatry.

[18] McHugo M, Talati P, Armstrong K, Vandekar SN, Blackford JU, Woodward ND (2019). Hyperactivity and reduced activation of anterior hippocampus in early psychosis. Am J Psychiatry.

[19] Schobel SA, Chaudhury NH, Khan UA, Paniagua B, Styner MA, Asllani I (2013). Imaging patients with psychosis and a mouse model establishes a spreading pattern of hippocampal dysfunction and implicates glutamate as a driver. Neuron.

[20] van Erp TG, Hibar DP, Rasmussen JM, Glahn DC, Pearlson GD, Andreassen OA (2016). Subcortical brain volume abnormalities in 2028 individuals with schizophrenia and 2540 healthy controls via the ENIGMA consortium. Mol Psychiatry.

[21] Fonville L, Drakesmith M, Zammit S, Lewis G, Jones DK, David AS (2019). MRI indices of cortical development in young people with psychotic experiences: influence of genetic risk and persistence of symptoms. Schizophr Bull.

[22] Schoorl J, Barbu MC, Shen X, Harris MR, Adams MJ, Whalley HC (2021). Grey and white matter associations of psychotic-like experiences in a general population sample (UK Biobank). Transl Psychiatry.

[23] Cao H, Cannon TD (2020). New evidence supporting a role of hippocampus in the development of psychosis. Biol Psychiatry.

[24] Haukvik UK, Tamnes CK, Soderman E, Agartz I (2018). Neuroimaging hippocampal subfields in schizophrenia and bipolar disorder: a systematic review and meta-analysis. J Psychiatr Res.

[25] Steen RG, Mull C, McClure R, Hamer RM, Lieberman JA (2006). Brain volume in first-episode schizophrenia: systematic review and meta-analysis of magnetic resonance imaging studies. Br J Psychiatry.

[26] Adriano F, Caltagirone C, Spalletta G (2012). Hippocampal volume reduction in first-episode and chronic schizophrenia: a review and meta-analysis. Neuroscientist.

[27] Calvo A, Roddy DW, Coughlan H, Kelleher I, Healy C, Harley M (2020). Reduced hippocampal volume in adolescents with psychotic experiences: a longitudinal population-based study. PLoS ONE.

[28] Kelleher I, Murtagh A, Molloy C, Roddy S, Clarke MC, Harley M (2012). Identification and characterization of prodromal risk syndromes in young adolescents in the community: a population-based clinical interview study. Schizophr Bull.

[29] Derome M, Zoller D, Modinos G, Schaer M, Eliez S, Debbane M (2020). Developmental trajectories of subcortical structures in relation to dimensional schizotypy expression along adolescence. Schizophr Res.

[30] Sahakyan L, Meller T, Evermann U, Schmitt S, Pfarr JK, Sommer J (2021). Anterior vs posterior hippocampal subfields in an extended psychosis phenotype of multidimensional schizotypy in a nonclinical sample. Schizophr Bull.

[31] Wood SJ, Kennedy D, Phillips LJ, Seal ML, Yucel M, Nelson B (2010). Hippocampal pathology in individuals at ultra-high risk for psychosis: a multi-modal magnetic resonance study. Neuroimage.

[32] Brunner G, Gajwani R, Gross J, Gumley AI, Krishnadas R, Lawrie SM (2022). Hippocampal structural alterations in early-stage psychosis: Specificity and relationship to clinical outcomes. Neuroimage Clin.

[33] Hinney B, Walter A, Aghlmandi S, Andreou C, Borgwardt S (2020). Does hippocampal volume predict transition to psychosis in a high-risk group? A meta-analysis. Front Psychiatry.

[34] Hu N, Luo C, Zhang W, Yang X, Xiao Y, Sweeney JA (2020). Hippocampal subfield alterations in schizophrenia: a selective review of structural MRI studies. Biomark Neuropsychiatry.

[35] Walter A, Suenderhauf C, Harrisberger F, Lenz C, Smieskova R, Chung Y (2016). Hippocampal volume in subjects at clinical high-risk for psychosis: a systematic review and meta-analysis. Neurosci Biobehav Rev.

[36] Ho NF, Holt DJ, Cheung M, Iglesias JE, Goh A, Wang M (2017). Progressive decline in hippocampal CA1 volume in individuals at ultra-high-risk for psychosis who do not remit: findings from the longitudinal youth at risk study. Neuropsychopharmacology.

[37] Provenzano FA, Guo J, Wall MM, Feng X, Sigmon HC, Brucato G (2020). Hippocampal pathology in clinical high-risk patients and the onset of schizophrenia. Biol Psychiatry.

[38] Ho NF, Iglesias JE, Sum MY, Kuswanto CN, Sitoh YY, De Souza J (2017). Progression from selective to general involvement of hippocampal subfields in schizophrenia. Mol Psychiatry.

[39] Lieberman JA, Girgis RR, Brucato G, Moore H, Provenzano F, Kegeles L (2018). Hippocampal dysfunction in the pathophysiology of schizophrenia: a selective review and hypothesis for early detection and intervention. Mol Psychiatry.

[40] Sasabayashi D, Yoshimura R, Takahashi T, Takayanagi Y, Nishiyama S, Higuchi Y (2021). Reduced hippocampal subfield volume in schizophrenia and clinical high-risk state for psychosis. Front Psychiatry.

[41] Kelleher I, Harley M, Murtagh A, Cannon M (2011). Are screening instruments valid for psychotic-like experiences? A validation study of screening questions for psychotic-like experiences using in-depth clinical interview. Schizophr Bull.

[42] Kaufman J, Birmaher B, Brent D, Rao U, Flynn C, Moreci P (1997). Schedule for affective disorders and schizophrenia for school-age children-present and lifetime version (K-SADS-PL): initial reliability and validity data. J Am Acad Child Adolesc Psychiatry.

[43] American Psychiatric Association. Diagnostic and statistical manual of mental disorders : DSM-5. 5th ed. Washington, DC: American Psychiatric Association; 2013. xliv, p. 947.

[44] Carey E, Gillan D, Healy C, Dooley N, Campbell D, McGrane J (2021). Early adult mental health, functional and neuropsychological outcomes of young people who have reported psychotic experiences: a 10-year longitudinal study. Psychological Med.

[45] Kelleher I, Cannon M. SOCRATES Assessment of Perceptual Abnormalities and Unusual Thought Content. 2014.

[46] Miller TJ, McGlashan TH, Rosen JL, Cadenhead K, Cannon T, Ventura J (2003). Prodromal assessment with the structured interview for prodromal syndromes and the scale of prodromal symptoms: predictive validity, interrater reliability, and training to reliability. Schizophr Bull.

[47] Calem M, Bromis K, McGuire P, Morgan C, Kempton MJ (2017). Meta-analysis of associations between childhood adversity and hippocampus and amygdala volume in non-clinical and general population samples. Neuroimage Clin.

[48] Hakamata Y, Suzuki Y, Kobashikawa H, Hori H (2022). Neurobiology of early life adversity: A systematic review of meta-analyses towards an integrative account of its neurobiological trajectories to mental disorders. Front Neuroendocrinol.

[49] Iglesias JE, Augustinack JC, Nguyen K, Player CM, Player A, Wright M (2015). A computational atlas of the hippocampal formation using ex vivo, ultra-high resolution MRI: application to adaptive segmentation of in vivo MRI. Neuroimage.

[50] Iglesias JE, Van Leemput K, Augustinack J, Insausti R, Fischl B, Reuter M (2016). Bayesian longitudinal segmentation of hippocampal substructures in brain MRI using subject-specific atlases. Neuroimage.

[51] R Core Team. R: A Language and Environment for Statistical Computing. Vienna, Austria: R Foundation for Statistical Computing; 2020.

[52] Bates D, Maechler M, Bolker B, Walker S (2015). Fitting linear mixed-effects models using lme4. J Stat Softw.

[53] Amaral DG (1978). A Golgi study of cell types in the hilar region of the hippocampus in the rat. J Comp Neurol.

[54] Gaarskjaer FB (1986). The organization and development of the hippocampal mossy fiber system. Brain Res.

[55] Allison PD. Handling missing data by maximum likelihood. SAS Global Forum; 2012.

[56] Zuur AF. Mixed effects models and extensions in ecology with R. New York, NY: Springer; 2009. xxii, p. 574.

[57] Benjamini Y, Hochberg Y (1995). Controlling the false discovery rate: a practical and powerful approach to multiple testing. JR Stat Soc B.

[58] Vasilopoulos T, Morey TE, Dhatariya K, Rice MJ (2016). Limitations of significance testing in clinical research: a review of multiple comparison corrections and effect size calculations with correlated measures. Anesth Analg.

[59] Sullivan GM, Feinn R (2012). Using effect size-or why the P value is not enough. J Grad Med Educ.

[60] Nakahara S, Matsumoto M, van Erp TGM (2018). Hippocampal subregion abnormalities in schizophrenia: a systematic review of structural and physiological imaging studies. Neuropsychopharmacol Rep.

[61] Vargas T, Dean DJ, Osborne KJ, Gupta T, Ristanovic I, Ozturk S (2018). Hippocampal subregions across the psychosis spectrum. Schizophr Bull.

[62] Ristanovic I, Vargas TG, Damme KSF, Mittal VA (2023). Hippocampal subfields, daily stressors, and resting cortisol in individuals at clinical high-risk for psychosis. Psychoneuroendocrinology.

[63] Mondelli V, Pariante CM, Navari S, Aas M, D’Albenzio A, Di Forti M (2010). Higher cortisol levels are associated with smaller left hippocampal volume in first-episode psychosis. Schizophr Res.

[64] Seabury RD, Cannon TD (2020). Memory impairments and psychosis prediction: a scoping review and theoretical overview. Neuropsychol Rev.

[65] Tamminga CA, Stan AD, Wagner AD (2010). The hippocampal formation in schizophrenia. Am J Psychiatry.

[66] Behrendt RP (2010). Contribution of hippocampal region CA3 to consciousness and schizophrenic hallucinations. Neurosci Biobehav Rev.

[67] Alkan E, Davies G, Greenwood K, Evans SLH (2020). Brain structural correlates of metacognition in first-episode psychosis. Schizophr Bull.

[68] Orfei MD, Piras F, Banaj N, Di Lorenzo G, Siracusano A, Caltagirone C (2017). Unrealistic self-overconfidence in schizophrenia is associated with left presubiculum atrophy and impaired episodic memory. Cortex.

[69] Gur RC, Calkins ME, Satterthwaite TD, Ruparel K, Bilker WB, Moore TM (2014). Neurocognitive growth charting in psychosis spectrum youths. JAMA Psychiatry.

[70] O’Neill A, Carey E, Dooley N, Healy C, Coughlan H, Kelly C (2020). Multiple network dysconnectivity in adolescents with psychotic experiences: a longitudinal population-based study. Schizophr Bull.

[71] Kelleher I, Cannon M (2021). A neural efficiency-threshold model to understand psychotic experiences. Psychol Med.

[72] Lynch KM, Shi Y, Toga AW, Clark KA, Pediatric Imaging N, Genetics S. (2019). Hippocampal Shape Maturation in Childhood and Adolescence. Cereb Cortex.

[73] Baglivo V, Cao B, Mwangi B, Bellani M, Perlini C, Lasalvia A (2018). Hippocampal subfield volumes in patients with first-episode psychosis. Schizophr Bull.

[74] Mohr C, Claridge G (2015). Schizotypy-do not worry, it is not all worrisome. Schizophr Bull.

[75] Coughlan H, Humphries N, Clarke MC, Healy C, Cannon M (2022). Psychotic-like experiences? Trajectories and typologies of hallucinations and delusions from early adolescence to early adulthood in a population-based sample of Irish youth. Ir J Psychol Med.

[76] Guloksuz S, van Os J (2018). The slow death of the concept of schizophrenia and the painful birth of the psychosis spectrum. Psychol Med.

[77] Wisse LEM, Chetelat G, Daugherty AM, de Flores R, la Joie R, Mueller SG (2021). Hippocampal subfield volumetry from structural isotropic 1 mm(3) MRI scans: A note of caution. Hum Brain Mapp.

