# Supplementary material for: Longitudinal hippocampal subfield development associated with psychotic experiences in young people
Source: Transl Psychiatry. 2024 Jan 20;14:44. doi: 10.1038/s41398-024-02746-w (PMC10799917; doi:10.1038/s41398-024-02746-w)
Supplement: Supplementary file 1 — Supplementary Material [file 41398_2024_2746_MOESM1_ESM.docx]

# Longitudinal hippocampal subfield development associated with psychotic experiences in young people

Aisling O'Neill^1,2,3^*, Niamh Dooley^2,3^, Darren Roddy^2,3^, Eleanor Carey^2^, Thomas Frodl^3,4^, Erik O’Hanlon^2,3^, Mary Cannon^2,3^

^1^Department of Psychology, St Patrick’s Mental Health Services, Dublin, Ireland

^2^Department of Psychiatry, RCSI University of Medicine and Health Sciences, St Stephens Green, Dublin, Ireland

^3^Trinity College Institute of Neuroscience, Trinity College Dublin, Dublin, Ireland

^4^Klinik für Psychiatrie, Psychotherapie und Psychosomatik, Uniklinik RWTH Aachen, Germany

***Corresponding author:** Dr Aisling O'Neill, Department of Psychiatry, RCSI University of Medicine and Health Sciences, St Stephens Green, Dublin, Ireland. Email: [aoneill@rcsi.com](mailto:aoneill@rcsi.com)

# Hippocampal subregions - Supplementary Material

## Childhood Adversity

As part of the K-SADS assessment at the initial assessment, participants and their parents/guardians were interviewed about any history of physical/sexual abuse relating to the adolescent, or any incidents of bullying victimisation relating to the adolescent. In the current sample, three participants reported both physical abuse and bullying. No participants reported any experiences of sexual abuse. As such, childhood adversity was treated as a dichotomous variable (0 = no childhood adversity reported, 1 = any report of physical and/or sexual abuse, and/or bullying rated 4/10 or higher). In relation to bullying, participants rated their level of distress in response to being bullied on a scale from 0 to 10 (0 = no distress). The median distress rating in those who were bullied was 4/10. This was used as a cut-off point, with ratings above 4/10 classified as an adverse exposure.

## Classification Criteria for Psychotic Experiences for the Adolescent Brain Development Study

The psychosis subsection of the K-SADS and the additional questions from the SOCRATES instrument investigate hallucinations and delusional thinking. All data reported in response to questions about hallucinatory or delusional experiences were rated according to the following categories:

**Definite Psychotic Experience:** The reported experience was definitely hallucinatory or delusional in nature

**Weak Psychotic Experience:** The reported experience had hallucinatory or delusional qualities but the perception, thought or belief was not strong or convincing enough to be classified as a definite psychotic experience

**Not a Psychotic Experience:** No evidence that the reported experience had any hallucinatory or delusion-like qualities

Participants who had at least one experience that was rated by consensus as being a Definite Psychotic Experience were classified as having experienced psychotic experiences for the study. Being rated as having a Weak Psychotic Experiences only was insufficient to be classified as having psychotic experiences in this study, and these participants were excluded from this analysis (N = 24 – of those participants who were scanned at least once). The table below gives examples of how experiences were defined for the purposes of rating.*

| HALLUCINATIONS |  |
| --- | --- |
| **Auditory Verbal Phenomena** | DEFINITE  Hearing one or more voices saying at least one word  Hearing whispering or indistinct voices at normal volume or shouting  Hearing name being called only if attributed to a delusional belief about another entity calling the name (e.g. a ghost)  Brief episodes of hearing own thoughts aloud when associated with delusional ideation and are either distressing or disorganising  WEAK  Brief episodes of hearing own thoughts aloud when not associated with delusional ideation and are neither distressing nor disorganising  NOT CLASSFIEID AS A PE  Hypnagogic and hypnopompic auditory verbal experiences |
| **Non-verbal Auditory Phenomena** | DEFINITE  Hearing non-verbal sounds that are experienced as distressing or disorganising  Hearing non-verbal sounds that are associated with delusional ideation  WEAK  Brief auditory experiences such as hearing music or other non-verbal sounds (e.g. hearing footsteps or knocking sounds) in the absence of any associated delusional ideation  NOT CLASSFIEID AS A PE  Hypnagogic and hypnopompic non-verbal auditory experiences |
| **Non-auditory Perceptual Phenomena** | DEFINITE  Seeing figures or forms that are not there (e.g. ghosts, human forms, aliens, the devil)  Tactile sensations that are associated with delusional attribution  WEAK  Recurrent experiences of smelling food that is not there  NOT CLASSFIEID AS A PE  Hypnagogic and hypnopompic visual experiences  Visual illusions  Occasional experiences of smells or tastes without any associated distress |
| DELUSIONS |  |
| **Unusual Thoughts and Beliefs** | DEFINITE  Delusional beliefs pertaining to any of the hallucinatory experiences in Domains 1-3  Definite and fixed beliefs about being watched by a person, entity or organisation  Recurrent and unfounded paranoid ideas that other people are criticising the individual  Beliefs that non-human entities (e.g. ghosts, spirits, aliens, the devil) are communicating directly with the individual  Unshakable nihilistic beliefs  Mind reading (self or others) when accompanied by paranoid beliefs that the individual has been singled out to have his/her mind read for a negative or nefarious purpose  WEAK  Vague sense or thought that individual is being watched  Mind reading (self or others) if not accompanied by a belief that the individual has been singled out to have his/her mind read for a negative or nefarious purpose  Magical thinking (e.g. a belief that the individual can predict the future) if not accompanied by distress or leading to disorganisation  NOT CLASSIFIED AS A PE  Thoughts and beliefs about being watched, judged or criticised by others that occur due to self-consciousness  A belief in ghosts, spirits or aliens are responsible or can influence experiences when such beliefs are aligned to normative cultural or subcultural beliefs  Subcultural beliefs that the world is coming to an end  Subcultural conspiracy beliefs |

* Exclusions: Hallucinatory experiences that occur in the context of an organic illness or that occur during acute intoxication

** Descriptions are not exhaustive

## DSM-5 Diagnoses

Table S1: Breakdown of lifetime DSM-5 diagnoses amongst those included in the neuroimaging study

| **Diagnosis** | **PE (n=22)** | **Controls (n=20)** |
| --- | --- | --- |
| Depressive disorder | 15 | 6 |
| Anxiety disorder | 7 | 3 |
| Eating disorder | 2 | 0 |
| Substance use disorder | 7 | 0 |
| Adjustment disorder | 2 | 0 |
| Attention disorder | 3 | 0 |
| Autism spectrum disorder | 1 | 0 |
| Number of participants with multiple diagnoses | 12 | 2 |
| No diagnoses | 0 | 12 |

Several PE participants and control participants had multiple diagnoses, thus the numbers in the table reflect how many reported a diagnosis in each category of disorder.

## Full linear mixed effects model

ROI ~ ICV + time + age + sex + handedness + CHA + DSM5 + group + group*time (random FX)

Where ROI = region of interest, ICV = intracranial volume, CHA = childhood adversity, time in months since baseline, age in months at baseline.

## Additional demographic analyses

As the population of interest in this case is the general population, the original ABD interview study was a community sample from north Dublin and Kildare which was similar to the Irish 2006 census in terms of nationality and socioeconomic status(Kelleher et al., 2012). The subsequent nested neuroimaging sample (100 participants) did not significantly differ from the non-imaged sample in terms of age, sex, handedness, presence of psychotic experiences, SES, personal psychiatric history or family psychiatric history(O'Hanlon et al., 2015).

For completeness, Chi-squared statistics were performed exploring gender, PE status, and socioeconomic status compared between those who were included in the current neuroimaging sample, and those from the initial ABD recruitment who were not included here. No significant differences were observed between the groups on these variables (PE: *X^2^* = 0.67, p = 0.41; SES: *X^2^* = 2.47, p = 0.78; gender: *X^2^* = 0.7, p = 0.4) (Table S2).

Table S2: Demographic data comparing participants from the original ABD study who were included in the current neuroimaging study, and those who were not included.

|  | **ABD participants included in current study (n=78)** | **ABD participants not included (n=133)** | **Statistics** |
| --- | --- | --- | --- |
|  | **Mean (SD)** | |  |
| Gender (%male) | 52.6 | 46.6 | *X*^2^ = 0.7, p = 0.4 |
| Socioeconomic status | 2.18 (0.92) | 2.37 (1.1) | *X^2^* = 2.47, p = 0.78 |
| PE status (% PE at some timepoint) | 42.3 | 36 | *X^2^* = 0.67, p = 0.41 |

Socioeconomic status was established via highest parental occupation level, categorized as follows: 1 = professional work, 2 = managerial and technical work, 3 = nonmanual work, 4 = skilled manual work, 5 = semiskilled work, 6 = unskilled work, 7 = unemployed. PE=psychotic experiences; SD=standard deviation.

PE status, SES, and gender were also compared for participants who took part in the neuroimaging arm, stratifying them according to drop-out time. No significant differences were observed between the participants who dropped out after baseline, those who dropped out after follow-up 1, and those who completed all 3 timepoints (PE: X^2^ = 0.56, p = 0.77; SES: X^2^ = 10, p = 0.26; gender: X^2^ = 1.079, p = 0.58) (Table S3).

To further explore any potential attrition bias, we performed exploratory analysis of variance statistics on the regions displaying significant differences in the LME analyses, wherein participants are stratified according to the drop-out time. Specifically, the mean volumes at baseline were calculated separately for those participants that dropped-out after baseline, those that dropped out after follow-up 1, and those that completed all three timepoints. The means of these groups were then compared (van Belle, Fisher, Heagerty, & Lumley, 2004). No significant between group differences were observed for the baseline volumes of the left hippocampus (F = 0.538, p = 0.59) or the right hippocampus (F = 1.85, p = 0.16). Similarly, the mean volumes at follow-up 1 were computed for all subjects that have data for that visit. The follow-up 1 means of those who completed the study and those who did not were then compared. Again, no significant between group difference was observed for the left hippocampus (t = -0.14, p = 0.89) or the right hippocampus (t = -0.59, p = 0.56) (Table S3).

These exploratory analyses evaluate whether the outcomes for the participants who dropped out appear to be different from those who completed the study. In this case, there appears to be no difference.

Table S3: Demographic data comparing participants included in the current neuroimaging study, stratified according to their drop-out time.

|  | **Post-baseline (n=18)** | **Post-follow-up 1 (n=26)** | **Completed (n=34)** | **Statistics** |
| --- | --- | --- | --- | --- |
|  | **Mean (SD)** | | |  |
| Gender (%male) | 44.4 | 50 | 58.8 | *X*^2^ = 1.079, p = 0.58 |
| Socioeconomic status | 2.14 (0.53) | 2.28 (1.18) | 2.13 (0.92) | *X^2^* = 10, p = 0.26 |
| PE status (% PE at some timepoint) | 38.9 | 38.5 | 47.1 | *X*^2^ = 0.56, p = 0.77 |
| Baseline left presubiculum vol. |  |  |  |  |
| Baseline right CA1 vol. |  |  |  |  |
| Baseline right CA2/3 vol. |  |  |  |  |
| Baseline right subiculum vol. |  |  |  |  |
| Follow-up 1 left presubiculum vol. | N/A |  |  |  |
| Follow-up 1 right CA1 vol. | N/A |  |  |  |
| Follow-up 1 right CA2/3 vol. | N/A |  |  |  |
| Follow-up 1 right subiculum vol. | N/A |  |  |  |

Post-baseline = participants who did not return after baseline; Post-follow-up 1 = participants who did not return after follow-up 1; Completed = participants who completed all three timepoints. Socioeconomic status was established via highest parental occupation level, categorized as follows: 1 = professional work, 2 = managerial and technical work, 3 = nonmanual work, 4 = skilled manual work, 5 = semiskilled work, 6 = unskilled work, 7 = unemployed. PE=psychotic experiences; SD=standard deviation.

Multicollinearity was assessed using the variance inflation factor (VIF). The predictors used in the final models demonstrated low correlation (i.e. VIF < 5) (Figure 1).

Figure 1: Graph demonstrating low correlation for the predictors used in the final models.

Table S4: Results of the mixed effects model analyses for hippocampal subfield volumes in the PE and control participants

|  | ICV | | Gender | | Age at BL | | Handedness | | Time since BL | | DSM5 diagnosis ever | | Group | | Group x Time since BL | |
| --- | --- | --- | --- | --- | --- | --- | --- | --- | --- | --- | --- | --- | --- | --- | --- | --- |
| GM volumes of interest | *B* (SE) | t, p | *B* (SE) | t, p | *B* (SE) | t, p | *B* (SE) | t, p | *B* (SE) | t, p | *B* (SE) | t, p | *B* (SE) | t, p | *B* (SE) | t, p |
| L presubiculum | 0.000084 (0.000036) | t = 2.33,  p = 0.023^1^ | -9.74 (12.37) | t = -0.79,  p = 0.43 | -0.26 (0.31) | t = -0.85,  p = 0.4 | 12.05 (10.73) | t = 1.12,  p = 0.26 | -0.045 (0.047) | t = -0.95,  p = 0.35 | -0.96 (2.44) | t = -0.39,  p = 0.69 | -23.69 (10.26) | t = -2.37,  p = 0.021^1^  (FDR = 0.1) | N/A | N/A |
| R CA1 | 0.00023 (0.000073) | t = 3.13,  p = 0.0026^1^ | -17.74 (25.58) | t = -0.69,  p = 0.49 | 1.15 (0.63) | t = 1.82,  p = 0.073^1^ | 25.31 (21.02) | t = 1.2,  p = 0.23 | 0.018 (0.09) | t = 0.2,  p = 0.84 | -3.11 (4.6) | t = -0.67,  p = 0.5 | 11.08 (21.02) | t = 0.53,  p = 0.6 | -0.37 (0.18) | t = -2.082,  p = 0.043^1^  (FDR = 0.21) |
| R CA2/3 | 0.000059 (0.00003) | t = 1.93,  p = 0.06^2^ | -3.27 (10.44) | t = -0.31,  p = 0.75 | 0.15 (0.25) | t = 0.57,  p = 0.57 | 22.93 (9.6) | t = 2.39,  p = 0.019^1^ | -0.039 (0.043) | t = -0.91,  p = 0.37 | 3.98 (2.18) | t = 1.82,  p = 0.075 | 11.04 (8.64) | t = 1.16,  p = 0.25 | -0.22 (0.085) | t = -2.58,  p = 0.013^1^  (FDR = 0.065^2^) |
| R subiculum | 0.000092 (0.000041) | t = 2.21,  p = 0.03^1^ | -21.04 (14.11) | t = -1.49,  p = 0.14 | 0.49 (0.35) | t = 1.41,  p = 0.16 | -24.46 (13.25) | t = -1.84,  p = 0.069^2^ | 0.063 (0.06) | t = 1.056,  p = 0.3 | -5.38 (3.1) | t = -1.73,  p = 0.089 | -24.07 (11.43) | t = -2.1,  p = 0.039^1^  (FDR = 0.19) | N/A | N/A |

^1^=Significant p value. ^2^ = trend level effect. PE = psychotic experience, L = left, R = right. *B* = estimate of the fixed effect coefficient, SE = standard error, BL = baseline, FDR = false discovery rate, N/A = not applicable – for left presubiculum and right subiculum, the fitted model did not include the Group x Time interaction effect. FDR-corrected *p* is reported for variables of interest significant at the uncorrected level; all other values displayed are uncorrected.

## References

Kelleher, I., Murtagh, A., Molloy, C., Roddy, S., Clarke, M. C., Harley, M., & Cannon, M. (2012). Identification and characterization of prodromal risk syndromes in young adolescents in the community: a population-based clinical interview study. *Schizophr Bull, 38*(2), 239-246. doi:10.1093/schbul/sbr164

O'Hanlon, E., Leemans, A., Kelleher, I., Clarke, M. C., Roddy, S., Coughlan, H., . . . Cannon, M. (2015). White matter differences among adolescents reporting psychotic experiences: A population-based diffusion magnetic resonance imaging study. *JAMA Psychiatry, 72*(7), 668-677. doi:10.1001/jamapsychiatry.2015.0137
